# Keep Calm and Dialyze On: Debunking the Myths of Peritoneal Dialysis Leaks

**DOI:** 10.1016/j.ekir.2024.07.022

**Published:** 2024-07-26

**Authors:** Susan McGrath, Arsh K Jain

**Affiliations:** 1 London Health Sciences Center, London, Ontario, Canada

See Clinical Research on Pages 2627 and 2727

Two papers in this edition of KIR explore the feasibility of urgent-start peritoneal dialysis (PD), addressing clinicians’ concerns about using PD catheters without an adequate “break-in” or healing period. The fears of leaks, infections, or poor clearance from low-volume urgent-start PD has led many clinicians to choose hemodialysis (HD) when patients need to start dialysis urgently. For patients destined for PD, this exposes them to an additional procedure with the potential for complications, including risks of infection. Further, treatment inertia is a risk after the stress of starting dialysis, with many patients opting to stay with HD. The International Society of Peritoneal Dialysis guidelines recommend a waiting period of at least 2 weeks before use^1^ to reduce the risk of mechanical complications. Understandably, this has caused concern that urgent-start PD (starting before 2 weeks) may have implications for catheter survival and complications.

The studies in this issue challenge this prevailing concern. Both have examined the use of “urgent-start” PD, commenced within 2 weeks of catheter insertion. Whereas Jin *et al.*^2^ define all patients <2 weeks from PD catheter insertion as urgent-start, Tsihlis *et al.*^3^ further subcategorize patients as urgent early-start (<72 hours) and elective early-start (72 hours–14 days). This split in urgent-start and early-start has been described previously^4^ and allows for a more specific assessment of complications and outcomes with the early use of a PD catheter. A retrospective review published in Peritoneal Dialysis International earlier this year examined urgent-start PD patients (<72 hours) versus urgent HD patients and found no difference in patient survival or dialysis-related infectious outcomes.^5^ Considering that the median time from insertion to catheter use in the Jin *et al.*^2^ study was 4 days, it is likely that participants who fit the definitions of both urgent- and early-start were included.

Tsihlis *et al.*^3^ assessed leak rates in urgent-start patients versus conventional-start patients (>14 days postinsertion) for both modified Seldinger catheter insertions and surgical catheter insertions. They found that, while leaks were more common in urgent-start patients (6.9% vs. 0.6%), this had no impact on outcomes such as peritonitis, catheter malposition or technique survival in the median follow-up period of 27.9 months. The rate of leaks quoted in the conventional-start group of the Tsihlis *et al.*^3^ study is remarkably low (0.6%) compared to pericatheter leak rates quoted in some studies.^6^, ^7^, ^8^ The reason for this low rate is not clear but could be due to a combination of insertion technique, and a relatively high hospital admission rate (possibly allowing for supine dialysis).

Jin *et al.*^2^ are to be congratulated for achieving the challenge of a multicenter randomized control trial in patients who need to start dialysis urgently. Subjects were randomized to 2 weeks of either urgent-start PD or HD, followed by all patients being maintained on PD. Although blinding is not possible due to the nature of the interventions, it is still a significant accomplishment with a low (6%) drop-out rate. The leak rate in the 2 groups was the same (3.4% vs. 3.4%) with no difference in patient survival, PD catheter survival, or peritonitis-free survival in the 1 year of follow-up.

We believe that the most important message from both studies is that, despite leaks occurring with early PD catheter use, peritonitis, catheter survival, and patient survival were not affected by early use of the PD catheter. If leaks could be managed without a need to transfer to HD and do not result in an increased incidence of infection, the perceived risk of using a PD catheter before full healing has occurred becomes quite minimal. Numerous small observational studies echo the results these studies have demonstrated; higher leak rates in urgent-starts, but better or comparable risks of infections, technique survival, and patient survival (Figure 1).

**Figure 1:**
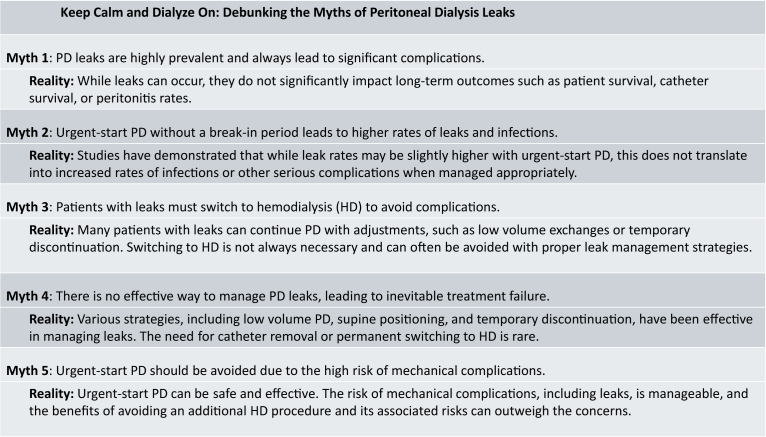
Keep calm and dialyze on: debunking the myths of peritoneal dialysis leaks.

Several strategies exist for managing leaks. In Tsihlis *et al.*,^3^ half of the patients with a leak continued PD with low volume continuous ambulatory PD or switching to automated PD. In 40% of the patients, PD was discontinued for 2 weeks then restarted. In 10% of the leaks, “bridging” HD was initiated, and PD was resumed after 6 weeks. The removal of a PD catheter was not required for any patients in the study; however, surgical management has been discussed in other papers.^9^ Another approach is to initiate low volume automated PD or a single icodextrin exchange in the supine position overnight. Further, some centers use antibiotics to reduce the theoretical risk of infection when a leak becomes evident.

Unfortunately, there is no standardized approach or robust evidence to inform leak management. What complicates management when patients require dialysis urgently, is that there is limited time to ensure the patient receives dialysis. Therefore, if there are leaks or other mechanical complications, clinicians often feel there is no time to hold dialysis or provide substandard therapy (e.g., low volume PD). Thus, clinicians readily default to HD or to providing bridging HD. Tsihlis *et al.*^3^ should be commended for avoiding HD in 90% of leaks. This evidence should provide reassurance to clinicians that in the majority of urgent dialysis cases, PD, even with a slightly slower start, is more than sufficient to manage cases.

A critical aspect of reporting on urgent-start PD is describing the insertion techniques in detail. These 2 papers vary in the data points reported. Jin *et al.*,^2^ for instance, do not report on their catheter insertion protocol. Tsihlis *et al.*^3^ reported the location of the deep cuff for surgical but not modified Seldinger insertions. Aspects of insertion technique such as a median versus paramedian approach, the use of rectus sheath tunneling or use of a deep stitch at the level of the anterior rectus sheath could impact leak rates. Similarly, though Jin *et al.*^2^ report the rate of heart failure in trial patients, liver failure or the presence of ascites are not reported by either study. The lack of consistent reporting of this data can limit effective analysis and comparison of research papers. Moreover, it makes it difficult for centers to understand why they may be experiencing differing outcome rates than those cited in the studies.

It is notable that, even in well-designed studies such as these, the reporting of data for many aspects of PD remains variable and unstructured. Data points on insertion technique, prescriptions and the management of complications can vary or are often not described. Therefore, standardizing this reporting will help with the application of the results, which is particularly important for papers dealing with the complications of a procedure. We must become more mindful of the details of different insertion approaches and their outcomes.

Despite this, the message from these studies is relatable to both surgical and modified Seldinger catheter insertions, inserted by nephrologists, interventional radiologists, or surgeons. Urgent-start or early-start PD in both studies showed low rates of early complications and no effect on long-term outcomes for patients. These studies provide compelling evidence that urgent-start PD is safe for patients, warranting a reconsideration of the practice of “bridging HD.”

## Disclosure

All the authors declared no competing interests.
